# Ontogeny and Stress-Induced Plasticity of Osmoregulatory Machinery in *Exopalaemon carinicauda*: Distinct Spatiotemporal Localization of Na^+^/K^+^-ATPase and Carbonic Anhydrase Isoforms

**DOI:** 10.3390/biology15171554

**Published:** 2026-09-05

**Authors:** Qianqian Ge, Jiajia Wang, Jian Li, Lu Wang, Jitao Li

**Affiliations:** 1 Laoshan National Laboratory, Qingdao 266237, China; ge2682@126.com; 2 State Key Laboratory of Mariculture Biobreeding and Sustainable Goods, Yellow Sea Fisheries Research Institute, Chinese Academy of Fishery Sciences, Qingdao 266071, China; jialp212@163.com (J.W.); bigbird@ysfri.ac.cn (J.L.)

**Keywords:** *Exopalaemon carinicauda*, Na^+^/K^+^-ATPase, carbonic anhydrase, gill, ontogeny, stress

## Abstract

The euryhaline shrimp *Exopalaemon carinicauda* is a valuable model for studying osmoregulation, yet the developmental establishment and spatial localization of its key ion-transport sites remain unclear. This study investigated the spatiotemporal expression of Na^+^/K^+^-ATPase (EcNKA), cytoplasmic carbonic anhydrase (EcCAc), and glycosylphosphatidylinositol-linked carbonic anhydrase (EcCAg) during early ontogeny and their subcellular responses to salinity and alkalinity stress in juvenile gills of *E. carinicauda*. Quantitative real-time PCR was used to detect the changes in mRNA expression of the three genes at different developmental stages, and immunohistochemistry was employed to determine the spatial localization of the corresponding three proteins. *EcNKA* and *EcCAg* mRNA expression peaked at zoea I, while *EcCAc* peaked at zoea III–IV. Immunostaining demonstrated a wide distribution of the three proteins across multiple tissues in early larvae, with a later restriction primarily to the gill and intestine. In gills, the EcNKA protein localized to septal cells, whereas EcCAc and EcCAg were located in pillar cells with distinct membrane polarities. Under low-salinity and high-alkalinity stress, transcriptional changes and protein-level responses of EcCAc and EcCAg were discordant. In addition, the protein location of EcCAc exhibited a translocation from the apical to the basolateral membrane, while EcCAg showed apical concentration under stress. These findings provide new insights into the ontogenetic establishment and subcellular plasticity of key ion-transport sites in *E. carinicauda* and help us to understand how euryhaline shrimp integrate developmental programming and saline–alkaline stress responses at both the mRNA and protein levels.

## Simple Summary

The ridgetail white prawn, *Exopalaemon carinicauda,* is a shrimp species that can live in coastal and inland saline–alkaline waters, making it an ideal subject for studying how aquatic animals maintain their osmotic balance. This study examined when and where three key ion-transport proteins (NKA, CAc and CAg) appear during the shrimp’s early development, and how they respond to low-salinity and high-alkalinity stress in juveniles. We found that these proteins become active at different developmental stages and gradually shift from being present throughout the body to becoming concentrated in the gills and intestines as the shrimp matures. When shrimp were exposed to low-salinity or high-alkalinity conditions, the amounts and locations of these proteins in their gills changed in complex ways that did not always match changes in their corresponding gene activities. These findings help us understand how shrimp adapt to saline–alkaline water conditions, which is valuable for improving shrimp farming practices and managing saline–alkaline aquaculture environments.

## 1. Introduction

The ridgetail white prawn, *Exopalaemon carinicauda*, is a euryhaline decapod widely distributed throughout the coastal and estuarine waters of the Yellow Sea and Bohai Sea [1,2]. Its remarkable tolerance to salinity (4–35‰) and alkalinity (≤13 mmol/L) make it an exemplary model for investigating the physiological and molecular underpinnings of saline–alkaline adaption in crustaceans [3,4]. In decapods, the gill epithelium serves as the primary interface for systemic ion homeostasis, housing an array of ion-transporting proteins and enzymes that coordinately drive active uptake or excretion of Na^+^, Cl^−^ and H^+^ equivalents to counter environmental osmotic challenges [5,6].

Among these transporters, Na^+^/K^+^-ATPase (NKA) is a crucial membrane-bound enzyme that establishes the primary driving force for transcellular ion transport by generating a Na^+^ gradient across the basolateral membrane [7,8]. Its pivotal role in salinity adaptation has been documented across numerous crustaceans, with both transcriptional upregulation and post-translational modulation of enzymatic activity reported under hypo- and hyper-osmotic conditions [9,10,11,12]. Carbonic anhydrases (CAs) constitute another critical component of the osmoregulatory machinery, catalyzing the reversible hydration of CO_2_ to HCO_3_^−^ and H^+^, thereby providing substrates for apical ion exchangers such as Na^+^/H^+^ and Cl^−^/HCO_3_^−^ antiporters [13]. In crustaceans, multiple CA isoforms exist, among which the cytoplasmic carbonic anhydrase (CAc) and membrane-associated glycosyl-phosphatidylinositol-linked carbonic anhydrase (CAg) isoforms are predominantly expressed in the gills [14]. Although both share a conserved α-CA catalytic domain, accumulating evidence suggests they play distinct functional roles, with CAc implicated in intracellular pH regulation and CAg more directly involved in transepithelial ion flux [15,16]. In *E. carinicauda*, the cDNAs encoding *EcNKA*, *EcCAc* and *EcCAg* have been cloned, all showing predominant expression in gills and significant responsiveness to salinity and alkalinity stress at the transcriptional levels [3,17]. Moreover, knockdown of *EcCAc* and *EcCAg* mRNA expression increased mortality under saline–alkaline stress, underscoring their indispensable physiological roles [3].

Despite these molecular advances, two substantial knowledge gaps remain that impede a comprehensive understanding of osmoregulation in *E. carinicauda*. First, the developmental trajectory of osmoregulatory competence remains entirely uncharted. In decapods, larvae initially rely on the general integument, antennal glands and branchiostegites for osmoregulation until the gill epithelium differentiates during zoea-to-postlarva metamorphosis [18,19]. For instance, in *Homarus gammarus*, NKA activity does not respond to dilute media until the postlarval stage, and CA activity is substantially higher in postlarvae than in earlier instars [20]. Similarly, in *Penaeus japonicus*, both NKA and CA activities peak in late postlarvae and adults [21]. Whether *E. carinicauda* follows a comparable developmental schedule and whether the two CA isoforms exhibit distinct temporal activation patterns during larval progression remain unknown. Elucidating this ontogenetic pattern is essential not only for understanding when active ion-transport capacity is established but also for interpreting stage-specific environmental tolerances from a comparative physiological perspective. Second, the cellular and subcellular localization of these ion-transporting proteins and their dynamic redistribution under osmotic stress have not been visualized. Previous transcriptional studies in shrimps have provided only bulk-tissue averages, leaving unresolved whether observed mRNA changes correspond to protein deployment in pillar cells, septal cells or specialized ionocytes and failing to capture potential post-translational events such as membrane trafficking [22,23,24]. This layer of regulation may be particularly critical, given that enzymatic activity often depends on conformational state and subcellular compartmentalization rather than transcript abundance alone [25]. Consequently, without knowledge of when the osmoregulatory site matures and where its components operate within the gills, the existing molecular data cannot be robustly linked to tissue-level physiology. Addressing these two interconnected gaps is therefore essential for a complete mechanistic model of saline–alkaline adaptation in this species.

The present study aimed to investigate the developmental establishment and spatial localization of osmoregulatory sites during post-embryonic development of *E. carinicauda* by characterizing the spatiotemporal expression of EcNKA, EcCAc and EcCAg, and to determine their functional roles in response to salinity and alkalinity stress by analyzing the transcriptional changes and subcellular redistribution of these three proteins in the gills. By combining quantitative real-time PCR (qRT-PCR) with immunohistochemistry (IHC), we sought to gain a holistic view of osmoregulatory adaptation by correlating developmental gene expression profiles with subcellular protein location, thereby providing a comprehensive basis for deciphering the salinity–alkalinity adaptation mechanism of *E. carinicauda*.

## 2. Materials and Methods

### 2.1. Animals and Experimental Design

All experimental shrimp were obtained from the same family line cultured in our laboratory. Three ovigerous females at the same brooding stage were selected, and each was reared individually in a separate 20 L aquarium (designated as biological replicate tanks A, B and C) containing aerated seawater at salinity 30‰, alkalinity 2 ± 0.3 mmol/L, temperature 24 ± 0.5 °C and pH 8.0 ± 0.2. Water quality was monitored daily, dissolved oxygen (DO) was maintained at 6.5 ± 0.3 mg/L via continuous aeration, and approximately 70% of the water was replaced weekly. After hatching, the larvae from each female were immediately transferred to new, identical aquaria under the same water quality conditions and fed with freshly hatched *Artemia nauplii* twice daily during the zoea stages. Larvae and postlarvae were sampled at the following early developmental stages: nauplius (N), pre-zoea (PZ), zoea I–V (ZI–ZV), and postlarva at day 5 (PL5). At each stage, six individuals were collected from each of the three replicate aquaria, pooled into one biological replicate, and snap-frozen in liquid nitrogen for subsequent RNA extraction (*n* = 3). Meanwhile, one individual from each of the three replicate aquaria was collected and fixed in 4% paraformaldehyde (PFA) for histological sectioning (*n* = 3).

After the remaining postlarvae were pooled and cultivated to the juvenile stage (body length 2.02 ± 0.25 cm), they were randomly divided into three treatment groups: control (C), low-salinity (LS), and high-alkalinity (HA). Each treatment comprised three parallel aquaria (*n* = 3 aquaria per treatment), with 20 juveniles per aquarium. For the LS group, water was exchanged once every three days, with an appropriate volume of freshwater added at each exchange to reduce salinity by 5‰ each time until it reached 5‰. Salinity was measured daily using a handheld refractometer (ATAGO, Tokyo, Japan) to confirm the target values. For the HA group, water was exchanged once every three days with NaHCO_3_ added to increase alkalinity by 2 mmol/L per exchange until it reached 12 mmol/L. Total alkalinity was determined daily by acid titration (HCl, 0.01 mol/L) according to standard seawater alkalinity assay methods. The water exchange frequency in the C group was identical. During the stress experiment, water temperature and dissolved oxygen were maintained at 24 ± 0.5 °C and 6.5 ± 0.3 mg/L, respectively. The pH remained at 8.0 ± 0.2 in the C and LS groups, while it increased to approximately 8.5 in the HA group due to the addition of NaHCO_3_. It should be noted that the pH rise is chemically coupled with alkalinity elevation, rendering these two factors non-separable confounders in the HA treatment. At 15 days post-treatment, gills from three juveniles in each aquarium were pooled into one mixed sample for RNA extraction (yielding 3 biological replicates per treatment, corresponding to the 3 aquaria, *n* = 3). Meanwhile, gills from one juvenile in each aquarium were dissected and fixed in 4% PFA for histological preparation (yielding 3 biological replicates per treatment, *n* = 3).

### 2.2. RNA Extraction and First-Strand cDNA Synthesis

Total RNA was extracted from whole individuals at each developmental stage using TRIzol reagent (Invitrogen, Carlsbad, CA, USA) according to the manufacturer’s protocol. RNA concentration and purity were assessed by spectrophotometry (NanoDrop 2000, Thermo Fisher Scientific, Waltham, MA, USA), and RNA integrity was verified by 1.5% agarose gel electrophoresis. Genomic DNA was removed by DNase I treatment. First-strand cDNA was synthesized from 1 μg of total RNA using the PrimeScript™ RT reagent Kit with gDNA Eraser kit (TaKaRa, Kusatsu, Shiga, Japan) following the manufacturer’s instructions. The resulting cDNA was stored at −20 °C until use.

### 2.3. Quantification of mRNA Expression by qRT-PCR

A commercial SYBR Green master mix (Vazyme Biotech, Nanjing, Jiangsu, China) was used for qRT-PCR on a LightCycler^®^ 480 II system (Roche, Basel, Switzerland). Each 20 μL reaction contained 10 μL of SYBR Green mix, 0.4 μL each of forward and reverse primers (10 μM), 2 μL of diluted cDNA template, and 7.2 μL of RNase-free water. The thermal cycling protocol was as follows: 95 °C for 30 s, followed by 40 cycles of 95 °C for 5 s and 60 °C for 30 s. Due to its stable expression, 18s rRNA was used as the internal reference gene [26]. The mRNA expression levels of target genes (*EcNKA*, *EcCAc*, *EcCAg*) were normalized to 18s rRNA (primer sequences are provided in Table S1). Relative expression was calculated using the 2^−ΔΔCt^ method. All reactions were performed in triplicate.

### 2.4. Preparation and Validation of the Specific Antibodies

A commercial rabbit polyclonal antibody against NKA (Catalog No. abs131657-50ug) was purchased from Absin Bioscience (Shanghai, China) and used for immunolocalization of EcNKA. The specificity of this antibody for EcNKA was confirmed by sequence alignment, which showed >85% similarity between the immunogen sequence and the EcNKA protein. The commercial anti-NKA antibody’s specificity in *E. carinicauda* was further validated by Western blotting using total protein extracts from gills. For EcCAc and EcCAg, specific rabbit polyclonal antibodies were raised using recombinant proteins expressed in *Escherichia coli*. Briefly, the open reading frames of *EcCAc* and *EcCAg* were amplified by PCR and cloned into pET-28a and pGS21a expression vectors (Takara, Kusatsu, Shiga, Japan), respectively (primer sequences are provided in Table S1). The recombinant plasmids were transformed into *E. coli* BL21 (DE3) competent cells (Yeasen, Shanghai, China). Protein expression was induced with 0.5 mM isopropyl β-D-1-thiogalactopyranoside (IPTG) at 28 °C for 12 h (EcCAc) or at 15 °C for 16 h (EcCAg). Recombinant proteins were purified using Ni^2+^-NTA columns (EcCAc) or GST affinity chromatography (EcCAg) (Yeasen, Shanghai, China). Purified proteins were injected into New Zealand rabbits to generate polyclonal antisera, and the specific antibodies were affinity-purified. Their specificity was validated by Western blotting (Sangon Biotech, Shanghai, China). The SDS-PAGE and Western blot analyses of EcCAc and EcCAg proteins are shown in Figure S1 and Figure S2, respectively.

### 2.5. Histological Preparations and Immunohistochemical Localization

From each individual, one representative paraffin section was selected for immunofluorescence staining. Specifically, fixed samples were dehydrated through a graded ethanol series, cleared in xylene, and embedded in paraffin wax. Serial sections (5 μm thickness) were cut using a microtome (Leica RM2235, Nussloch, Germany) and mounted onto poly-L-lysine-coated slides. For immunofluorescence, sections were deparaffinized in two changes of xylene (10 min each), rehydrated through a graded ethanol series (100%, 95%, 80%, and 70% ethanol, 5 min each), and washed in phosphate-buffered saline (PBS, pH 7.4) for 5 min. Heat-induced antigen retrieval was performed by immersing sections in EDTA retrieval buffer (pH 8.0) and microwaving at high power until boiling and then at medium power for 20 min. The sections were then allowed to cool naturally to room temperature. After three washes with PBS (5 min each) on a shaker, non-specific binding was blocked with 3% bovine serum albumin (BSA) in PBS for 30 min at room temperature. Sections were then incubated overnight at 4 °C in a humidified chamber with primary antibodies diluted in PBS containing 1% BSA: rabbit anti-NKA polyclonal antibody at 1:200 dilution; rabbit anti-EcCAc polyclonal antibody at 1:100 dilution; and rabbit anti-EcCAg polyclonal antibody at 1:200 dilution. After three PBS washes (5 min each), sections were incubated with fluorescence-conjugated secondary antibodies (goat anti-rabbit IgG) for 50 min at room temperature in the dark, followed by three PBS washes. Nuclei were counterstained with DAPI for 10 min at room temperature in the dark. After three additional PBS washes, spontaneous tissue autofluorescence was quenched by applying an autofluorescence quencher for 5 min, followed by a 10 min rinse in running tap water. Finally, sections were mounted with an anti-fade mounting medium. Negative controls were performed by replacing the primary antibody with pre-immune serum to confirm antibody specificity. Fluorescent images were captured using a fluorescence microscope (Zeiss, Oberkochen, Germany).

### 2.6. Image Analysis Procedure

Immunofluorescence images of gill sections were analyzed using ImageJ 1.54 software (National Institutes of Health, Bethesda, MD, USA). For each treatment group, three biological replicates (*n* = 3) were used for IHC analysis. From each biological replicate, one representative paraffin section of the gill tissue was selected for immunofluorescence staining and imaging. For each section, 3–5 randomly selected gill lamellae were captured as digital images. A total of 9–15 gill lamellae were analyzed per treatment group (3 animals × 3–5 lamellae). The fluorescence intensity and relative fluorescence area from these lamellae were averaged per animal, and the individual animal means (*n* = 3 per treatment) were used for statistical comparisons among groups. Briefly, each digital image was converted to an 8-bit grayscale, and the region of interest (ROI) was delineated using the polygon tool. For each ROI, the software automatically calculated the area, integrated density, and mean gray value. An adjacent region with no specific fluorescence was used to measure the background signal. The corrected mean fluorescence intensity was calculated as follows: (mean gray value of ROI)-(mean gray value of background). The relative fluorescence area was expressed as the percentage of the ROI area showing positive staining relative to the total ROI area.

### 2.7. Statistical Analysis

All data are presented as arithmetic means ± standard deviations (SDs). Statistical analyses were performed using GraphPad Prism software (version 8.0) and SPSS (version 22.0, IBM). For the comparisons of developmental stage and immunofluorescence quantification, one-way ANOVA followed by Tukey’s HSD post hoc test was used to perform all pairwise comparisons. For gene expression comparisons between the C group and each stress group (LS or HA), Welch’s corrected unpaired two-tailed Student’s *t*-tests were used. Statistical significance was set at *p* < 0.05.

## 3. Results

### 3.1. EcNKA, EcCAc and EcCAg mRNA Expression in Early Developmental Stages

**Figure 1:**
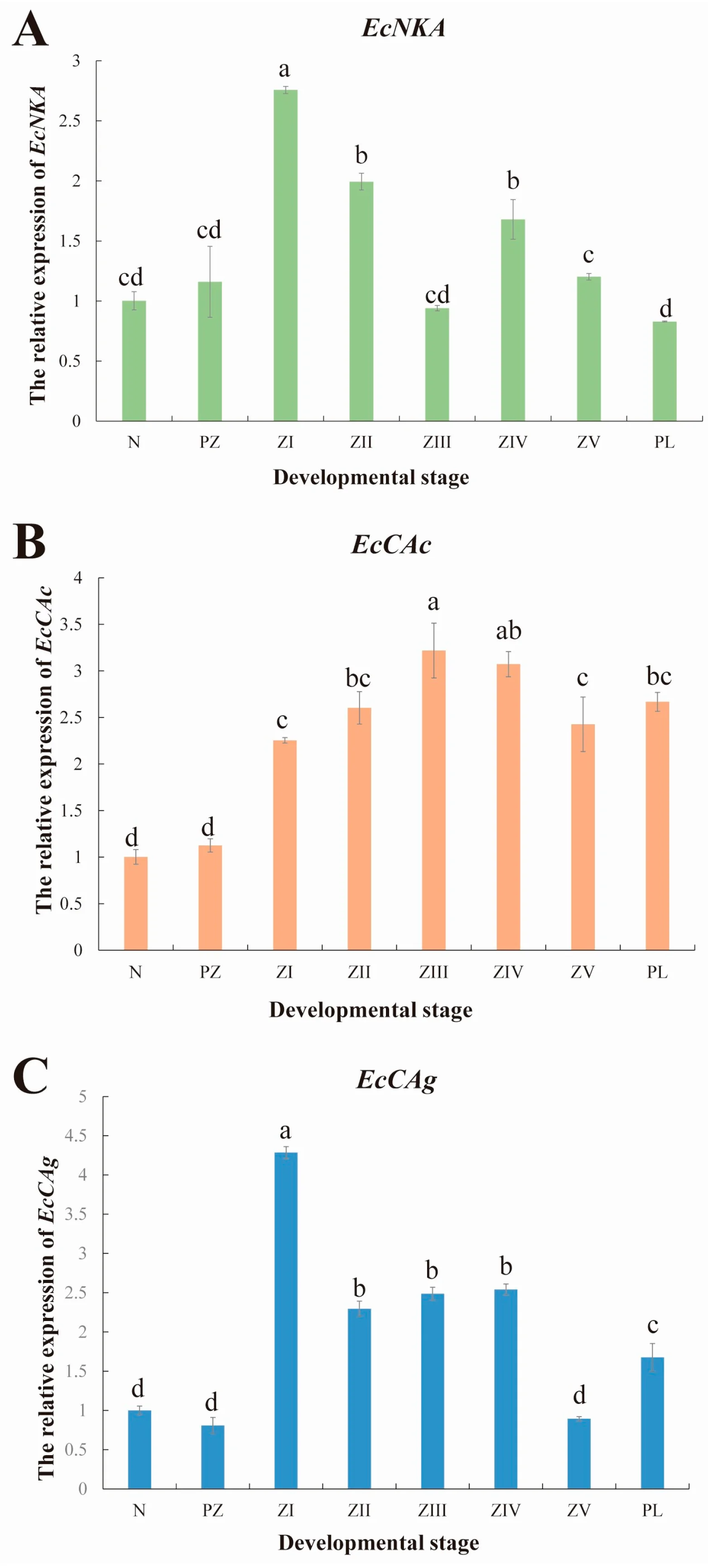
*EcNKA* (**A**), *EcCAc* (**B**) and *EcCAg* (**C**) mRNA expression in early developmental stages. Data are presented as means ± SDs. *n* = 3 biological replicates (aquaria) per stage, with six individuals pooled per replicate. Different letters indicate significant differences among different developmental stages (*p* < 0.05).

As shown in Figure 1, the mRNA expression levels of *EcNKA*, *EcCAc* and *EcCAg* generally exhibited a similar expression pattern during the larval and postlarval developmental stages of *E. carinicauda*, all showing an initial increase followed by a decrease. Among them, *EcNKA* and *EcCAg* exhibited low expression levels at the N and PZ stages, rapidly peaked at the ZI stage, and then significantly decreased (Figure 1A, F (7, 16) = 82.189, *p* < 0.0001; Figure 1C, F (7, 16) = 455.787, *p* < 0.0001). In contrast, *EcCAc* showed the lowest expression levels at the N and PZ stages and gradually increased to the highest levels at the ZIII and ZIV stages, followed by a subsequent decrease (Figure 1B, F (7, 16) = 66.978, *p* < 0.0001).

### 3.2. Specificity Validation of the EcCAc and EcCAg Polyclonal Antibodies

The commercial anti-NKA polyclonal antibody detected a single band at approximately 110 kDa, consistent with the expected molecular weight of the crustacean NKA α-subunit, confirming its specific recognition of EcNKA (Figure S3A). The ELISA signals of the purified EcCAc and EcCAg polyclonal antibodies were substantially higher than those of the pre-immune serum, confirming successful generation of specific antibodies against the target antigen with negligible non-specific binding (Tables S2 and S3). Western blotting demonstrated high specificity for both antibodies. Total proteins isolated from the gills were used for the detection of EcCAc and EcCAg antibodies, which showed two major protein bands of 29 kDa and 32.68 kDa (Figure S3B,C).

### 3.3. Protein Localization of EcNKA, EcCAc and EcCAg Proteins in Early Developmental Stages

**Figure 2:**
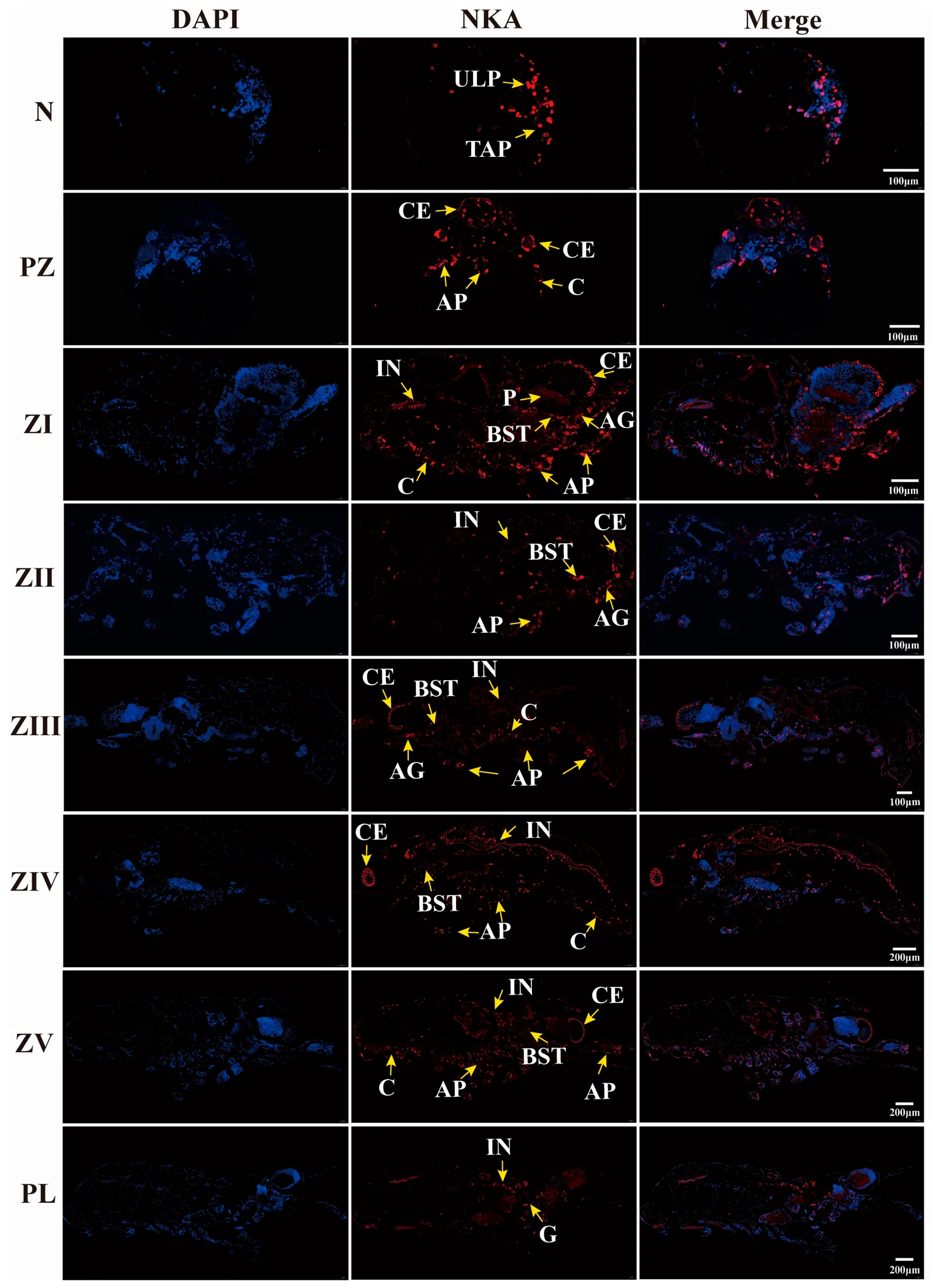
Immunolocalization of EcNKA in early developmental stages of *E. carinicauda*. ULP, upper lip primordium; TAP, thoracic and abdominal primordia; CE, compound eye; AP, appendage; IN, intestine; G, gill; P, pleura; AG, antennal gland; BST, branchiostegite; C, cuticle.

During the ontogeny of *E. carinicauda*, protein localization of EcNKA displayed dynamic spatial patterns. The immunostaining (IS) signals were confined to the labrum and thoracoabdominal primordia at stage N. The IS signals shifted to the compound eyes and appendage buds at the PZ stage. From the ZI to ZV stages, consistent EcNKA labeling was widely distributed in the compound eyes, pleura, branchiostegite, antennal gland, intestine, cuticle and appendages. Notably, the predominant EcNKA signals became localized mainly in the gills and intestine, whereas other tissues showed diminished or absent staining at the PL5 stage (Figure 2).

**Figure 3:**
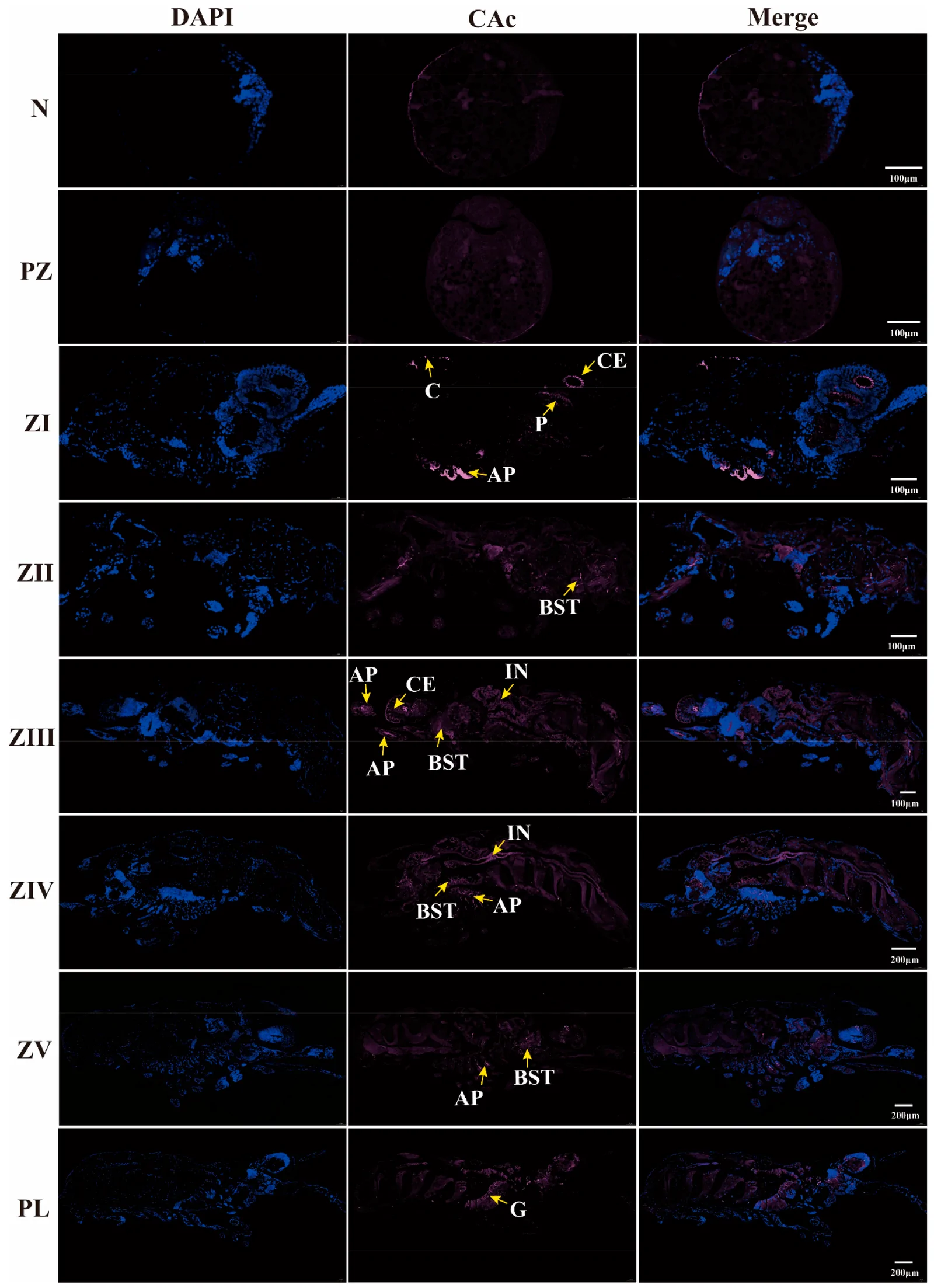
Immunolocalization of EcCAc in early developmental stages of *E. carinicauda*. CE, compound eye; AP, appendage; IN, intestine; G, gill; P, pleura; BST, branchiostegite; C, cuticle.

The protein location of EcCAc was barely detectable during the N and PZ stages. The IS signals were predominantly localized in the compound eyes, pleura and appendages at the ZI stage. From the ZII to ZV stages, EcCAc-positive signals were relatively evenly distributed throughout the body without being particularly concentrated in specific areas. The signals were primarily concentrated in the gills at the PL5 stage (Figure 3).

**Figure 4:**
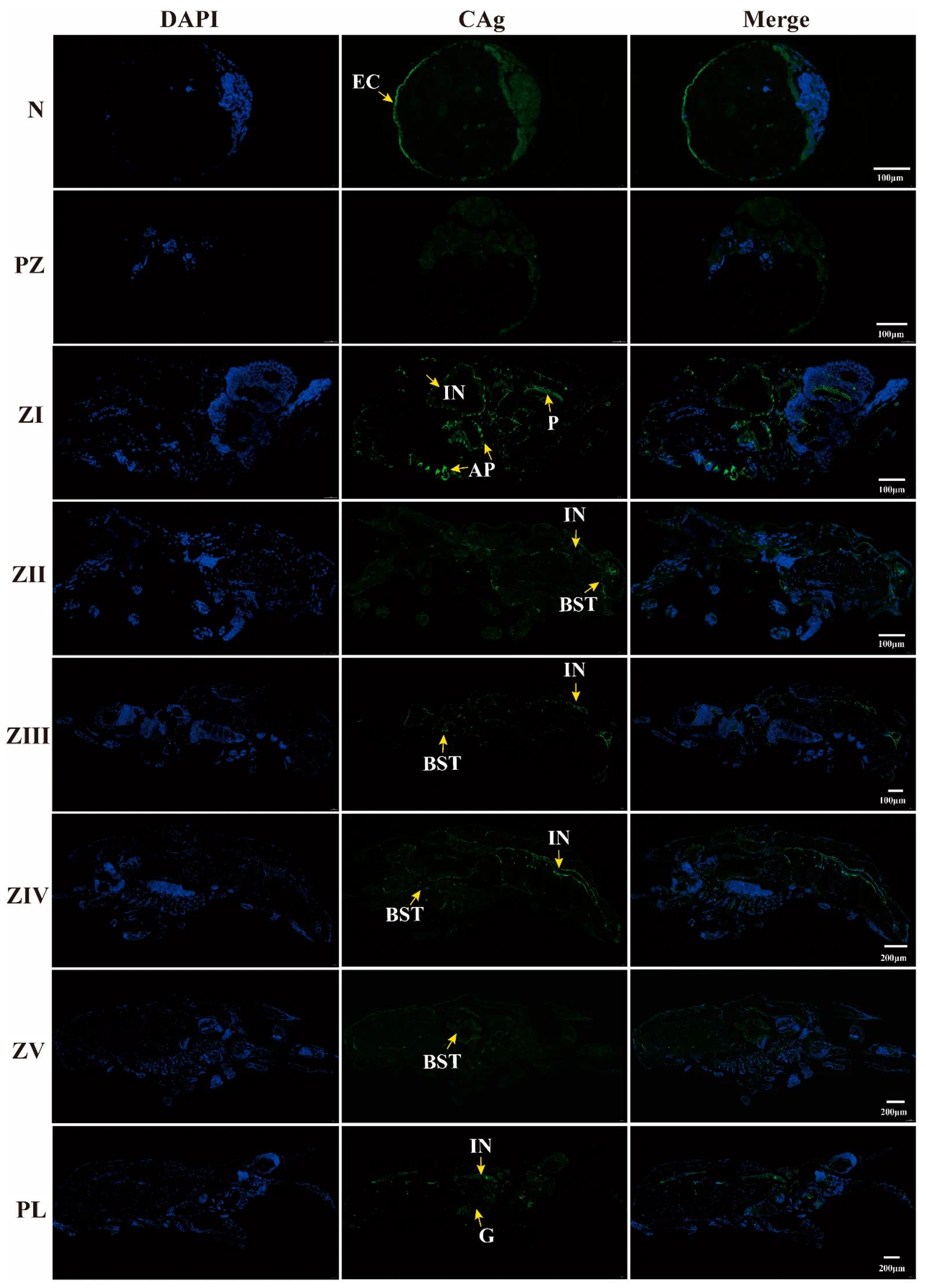
Immunolocalization of EcCAg in early developmental stages of *E. carinicauda*. CE, compound eye; AP, appendage; IN, intestine; G, gill; P, pleura; EC, embryonic cuticle; BST, branchiostegite; C, cuticle.

The protein location of EcCAg was mainly distributed in the cuticle at the N stage, whereas no obvious aggregated positive signals were detected at the PZ stage. At the ZI stage, signals were predominantly localized in the pleura, intestine and appendages. From the ZII to ZV stages, IS signals were detected mainly in the intestine and the branchial region. At the PL5 stage, signals were primarily distributed in the gills and intestine (Figure 4).

### 3.4. EcNKA, EcCAc and EcCAg mRNA Expression in Gills Under Salinity and Alkalinity Stress

**Figure 5:**
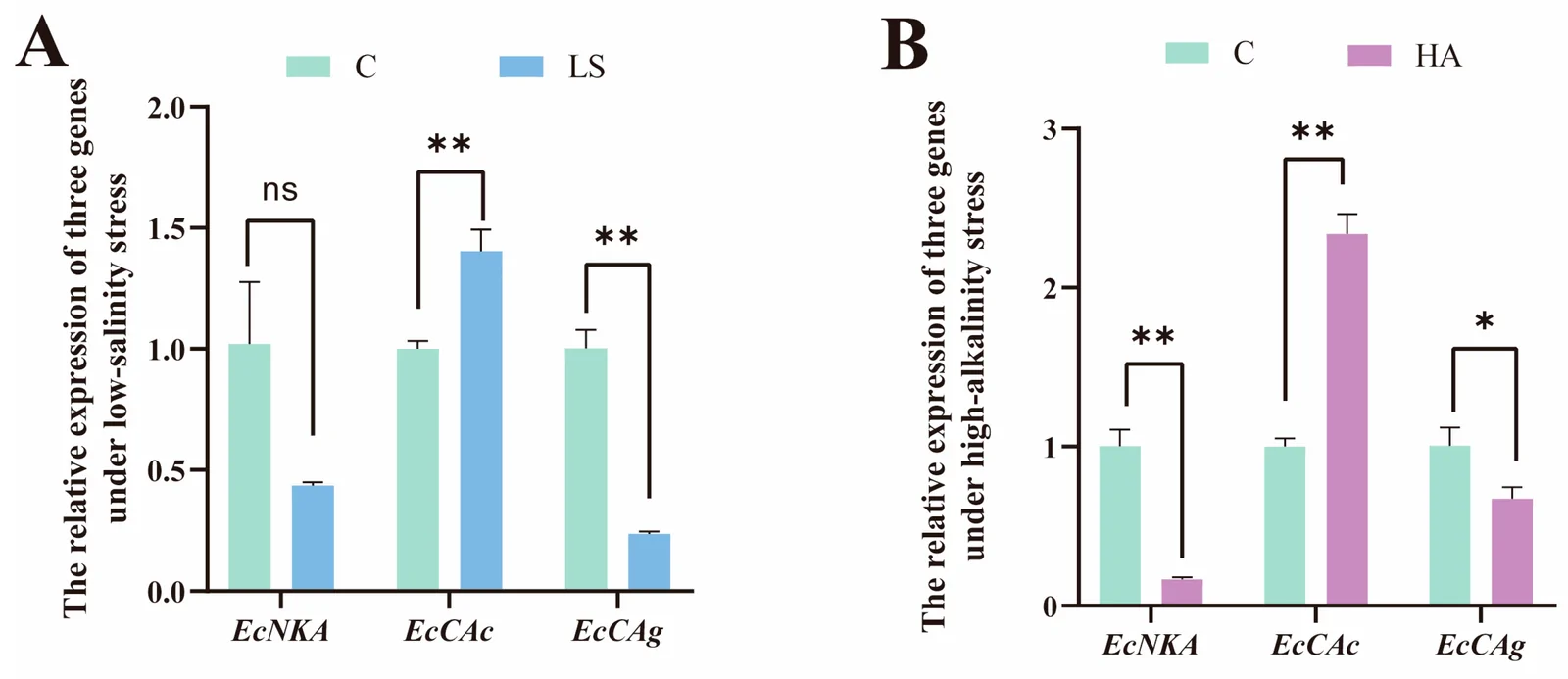
*EcNKA*, *EcCAc* and *EcCAg* mRNA expression under low-salinity (**A**) and high-alkalinity (**B**) stress. Data are presented as means ± SDs. *n* = 3 biological replicates (aquaria) per treatment, with gill tissues from three individuals pooled per replicate. Significance is indicated as follows: * *p* < 0.05; ** *p* < 0.01; ns, not significant.

Compared with the control group, the expression of *EcNKA* was significantly downregulated under high-alkalinity stress but showed no significant change under low-salinity stress (Figure 5A: *EcNKA*, t = 3.973, df = 2.013, *p* = 0.0573; Figure 5B: *EcNKA*, t = 13.99, df = 2.048, *p* = 0.0046). The *EcCAg* mRNA in the gills was significantly downregulated, while EcCAc was significantly upregulated after both low-salinity and high-alkalinity stress (Figure 5A: *EcCAc*, t = 7.346, df = 2.553, 0.0088; *EcCAg*, t = 17.06, df = 2.061, *p* = 0.0030; Figure 5B: *EcCAc*, t = 17.01, df = 2.631, *p* = 0.0009; *EcCAg*, t = 4.235, df = 3.364, *p* = 0.0191).

### 3.5. Localization of EcNKA, EcCAc and EcCAg Proteins in Gills Under Salinity and Alkalinity Stress

**Figure 6:**
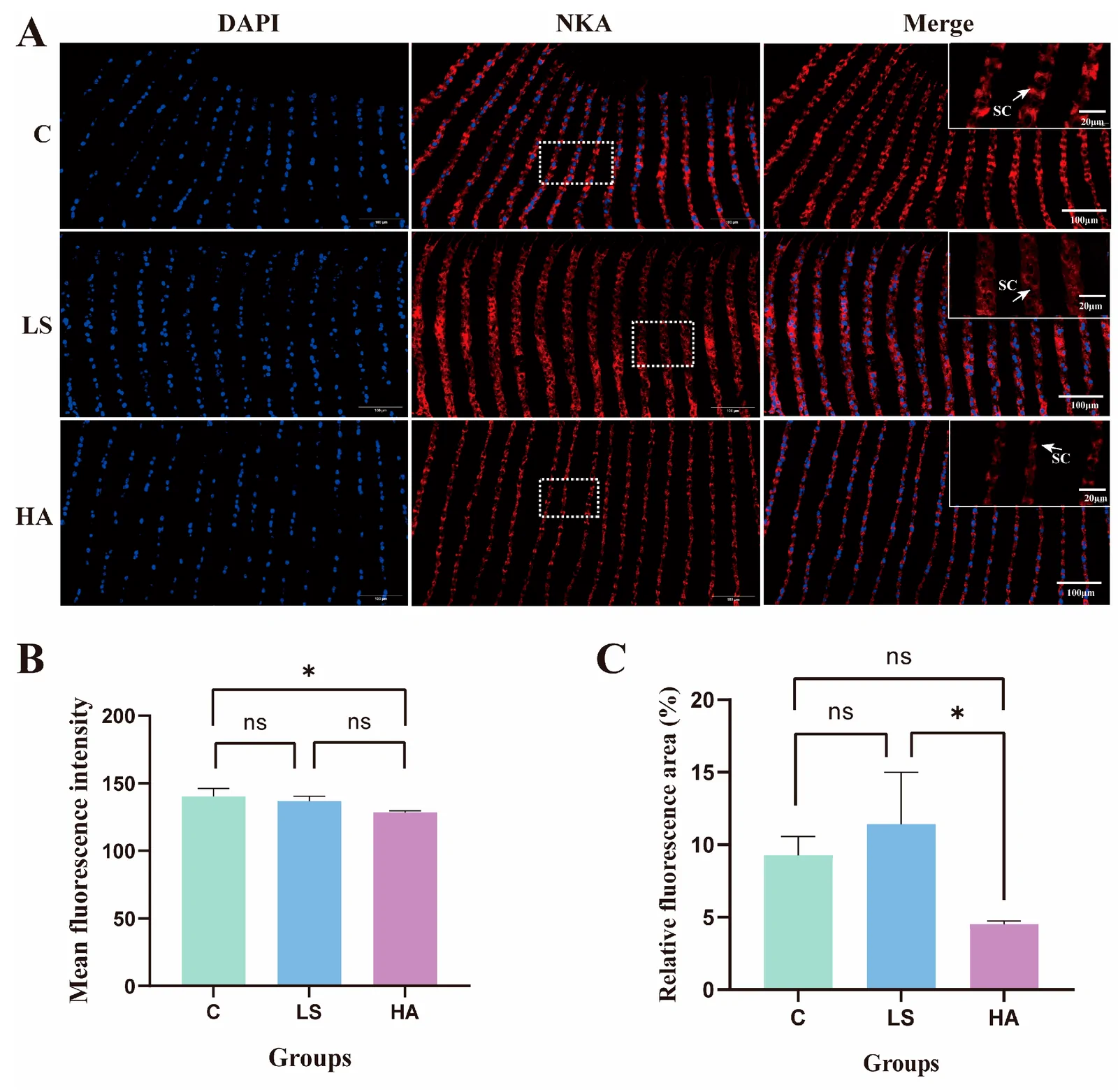
Immunolocalization of EcNKA in the gills of *E. carinicauda* under low-salinity and high-alkalinity stress. (**A**) Positive immunostaining of EcNKA under low-salinity and high-alkalinity stress. The figure in the upper right corner is an enlarged view. SC, septal cell. (**B**) The mean fluorescence intensity of EcNKA. (**C**) The relative fluorescence area (%). For statistical analysis, *n* = 3 biological replicates (aquaria) per treatment, with one individual per replicate. Bars and error bars represent means ± SDs of the three biological replicates. The significance is indicated as follows: * *p* < 0.05; ns, not significant.

EcNKA immunohistochemical analysis revealed that IS signals were predominantly localized in septal cells in the control group (Figure 6A). In the low-salinity group, the relative fluorescence area and fluorescence intensity did not change significantly. In the high-alkalinity group, the fluorescence intensity decreased substantially compared with the control (*p* < 0.05), while relative fluorescence area did not change significantly (Figure 6B, F (2, 6) = 6.890, *p* (C vs. LS) = 0.5547, *p* (C vs. HA) *=* 0.0259, *p* (LS vs. HA) = 0.0983; Figure 6C, F (2, 6) = 7.766, *p* (C vs. LS) = 0.4943, *p* (C vs. HA) *=* 0.0844, *p* (LS vs. HA) = 0.0198).

**Figure 7:**
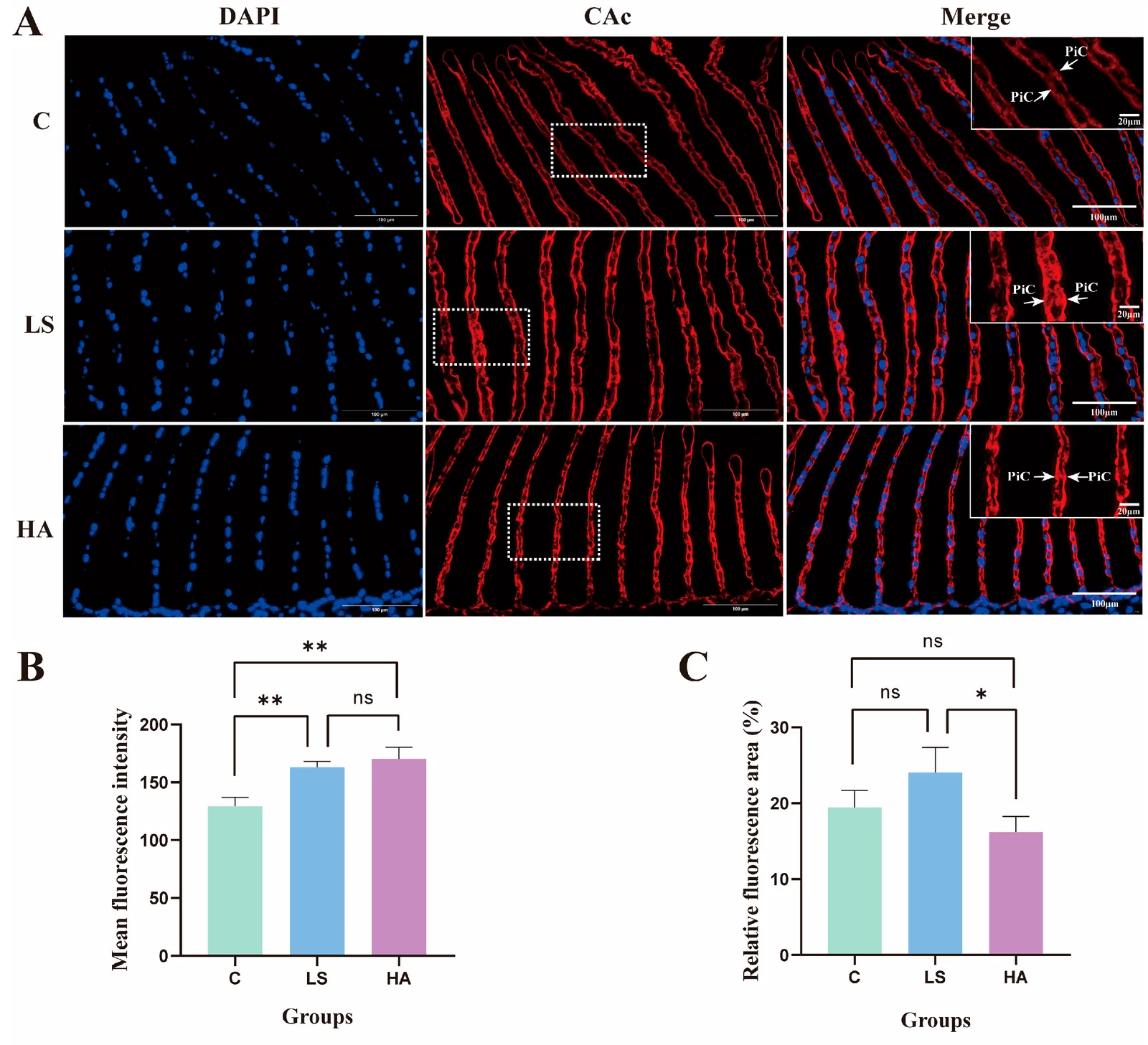
Immunolocalization of EcCAc in the gills of *E. carinicauda* under low-salinity and high-alkalinity stress. (**A**) Positive immunostaining of EcCAc under low-salinity and high-alkalinity stress. The figure in the upper right corner is an enlarged view of the square in the middle picture. PiC, pillar cell. (**B**) The mean fluorescence intensity of EcCAc. (**C**) The relative fluorescence area (%). For statistical analysis, *n* = 3 biological replicates (aquaria) per treatment, with one individual per replicate. Bars and error bars represent means ± SDs of the three biological replicates. The significance is indicated as follows: * *p* < 0.05; ** *p* < 0.01; ns, not significant.

In the control group, EcCAc expression was mainly confined to the apical membrane of pillar cells (Figure 7A). After low-salinity stress, the IS signal shifted toward the basal membrane of pillar cells, accompanied by an increase in fluorescence intensity, but with no significant change in relative fluorescence area. After high-alkalinity stress, a portion of the IS signal also shifted from the apical to the basal membrane of the pillar cells, and mean fluorescence intensity significantly increased (*p* < 0.05), while relative fluorescence area did not change significantly (Figure 7B, F (2, 6) = 22.71, *p* (C vs. LS) = 0.0048, *p* (C vs. HA) *=* 0.0018, *p* (LS vs. HA) = 0.5505; Figure 7C, F (2, 6) = 6.985, *p* (C vs. LS) = 0.1548, *p* (C vs. HA) *=* 0.3367, *p* (LS vs. HA) = 0.0230).

**Figure 8:**
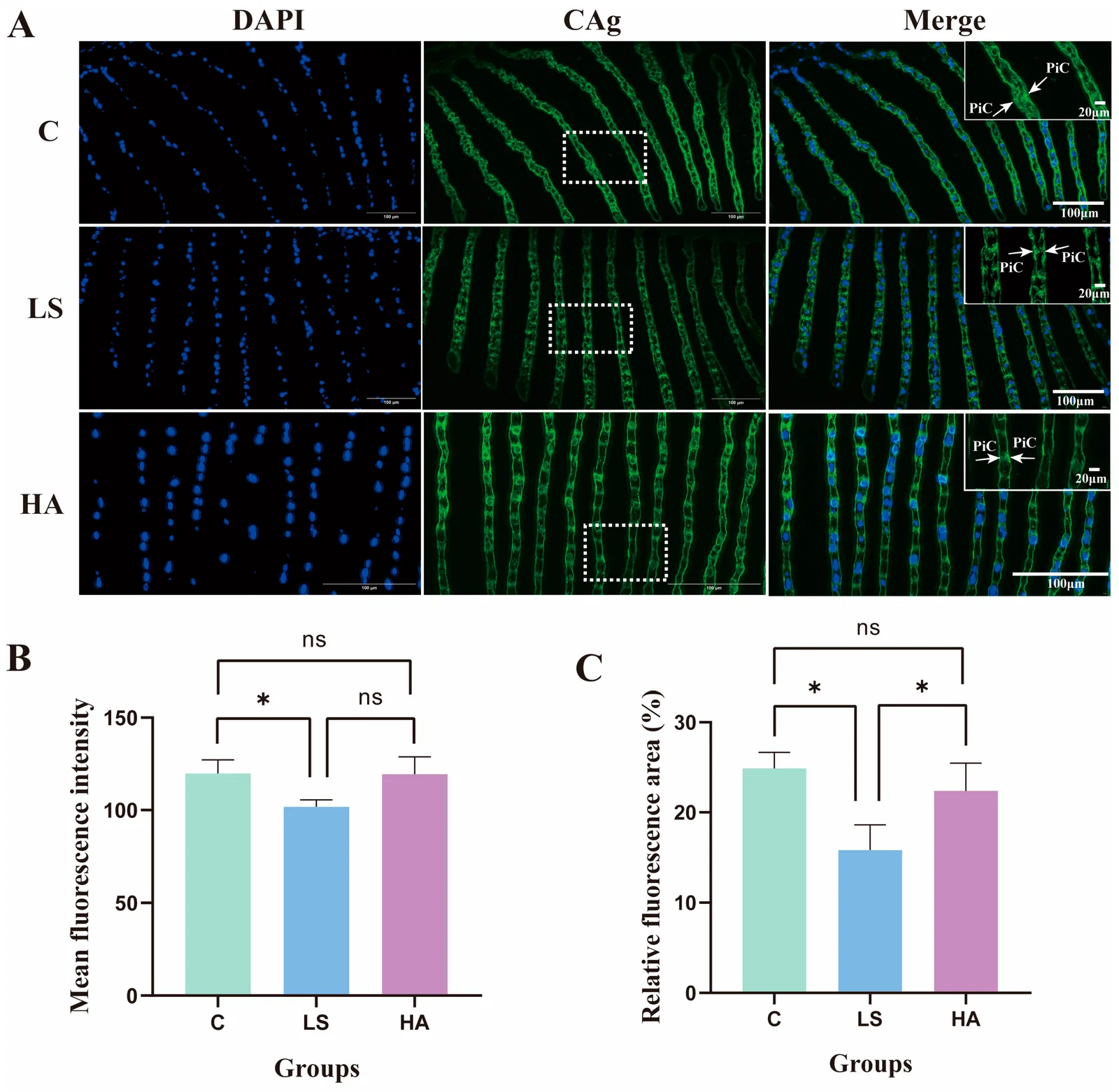
Immunolocalization of EcCAg in the gills of *E. carinicauda* under low-salinity and high-alkalinity stress. (**A**) Positive immunostaining of EcCAg under low-salinity and high-alkalinity stress. The figure in the upper right corner is an enlarged view of the square in the middle picture. PiC, pillar cell. (**B**) The mean fluorescence intensity of EcCAg. (**C**) The relative fluorescence area (%). For statistical analysis, *n* = 3 biological replicates (aquaria) per treatment, with one individual per replicate. Bars and error bars represent means ± SDs of the three biological replicates. The significance is indicated as follows: * *p* < 0.05; ns, not significant.

In the control group, EcCAg-positive signals were evenly distributed throughout pillar cells, including the cell membrane and cytoplasm. After both low-salinity and high-alkalinity stress, signals became concentrated at the apical membrane of pillar cells (Figure 8A). Mean fluorescence intensity and relative fluorescence area both decreased significantly after low-salinity stress, while they showed no significant change after high-alkalinity stress (Figure 8B, F (2, 6) = 6.145, *p* (C vs. LS) = 0.0499, *p* (C vs. HA) *=* 0.9973, *p* (LS vs. HA) = 0.0544; Figure 8C, F (2, 6) = 9.593, *p* (C vs. LS) = 0.0129, *p* (C vs. HA) *=* 0.5160, *p* (LS vs. HA) = 0.0494).

## 4. Discussion

The present study provides the first comprehensive spatiotemporal characterization of three key osmoregulatory proteins (EcNKA, EcCAc and EcCAg) during early ontogeny of *E. carinicauda* and further investigates their subcellular responses in juvenile gills to low-salinity and high-alkalinity challenges. Our data reveals that the osmoregulation machinery becomes functionally established during the zoea stages, with a subsequent shift in protein expression from widespread integumentary tissues to the gill epithelium during the postlarval transition. In addition, salinity and alkalinity stresses elicit not merely quantitative changes in gene expression but also differential membrane trafficking of these proteins within specific gill cell types, highlighting a previously underappreciated layer of post-translational regulation in crustacean osmoregulation.

### 4.1. Ontogeny of Osmoregulatory Capacity in E. carinicauda

In decapod crustaceans, the transition from larval to postlarval life is typically accompanied by a major reorganization of ion-transporting epithelia, with the gills gradually assuming the primary role in hemolymph ion homeostasis [12,27]. Our developmental expression profiles of *EcNKA*, *EcCAc* and *EcCAg* mRNA exhibited a general trajectory of first increasing and then decreasing from the nauplius to postlarva, yet the timing of peak expression differed among the three genes. *EcNKA* and *EcCAg* mRNA expression peaked as early as ZI, whereas EcCAc reached its maximum at ZIII-ZIV. This temporal divergence strongly suggests that the two CA isoforms are recruited at distinct phases of larval maturation. The early surge in *EcNKA* and *EcCAg* at ZI coincides with the transition from embryonic to free-swimming life, when larvae first encounter fluctuating external ion compositions. In the absence of fully differentiated gills, the widespread distribution of EcNKA immunoreactivity in the compound eyes, pleura, antennal glands and appendage buds at ZI-ZV indicates that osmoregulatory functions are distributed across multiple epithelia. This pattern is consistent with observations in *P. japonicus*, *H. gammarus* and *Macrobrachium amaonicum*, where NKA activity in whole larvae increases markedly during the zoea stages before gill differentiation [28,29,30]. The later peak of *EcCAc* expression at ZIII-ZIV may reflect a more sustained role in intracellular pH regulation and metabolic CO_2_ hydration, as larvae grow in size and their respiratory and excretory demands intensify [3,31,32].

At the postlarval stage, EcNKA, EcCAc and EcCAg immunoreactivities all became predominantly restricted to the gills and intestine, with marked diminution in other tissues. This spatial restriction is functionally analogous to the post-metamorphic gill differentiation observed in other euryhaline decapods, such as *Macrobrachium rosenbergii*, *Carcinus maenas* and *Crangon crangon*, whose gill epithelia acquire specialized ionocytes only after the metamorphic molt [33,34,35]. We therefore propose that the osmoregulatory capacity of *E. carinicauda* is progressively established during the zoea stages, and upon reaching the postlarval stage, the gill tissues undergo full maturation, serving as the primary osmoregulatory organs in this species. This developmental schedule should be considered when interpreting salinity and alkalinity tolerances at different life stages of *E. carinicauda*.

### 4.2. Functional Differentiation of CA Isoforms and Evolutionary Implications

Carbonic anhydrases are ubiquitously expressed in crustacean gills, but the functional distinction between CAc and CAg isoforms has remained ambiguous [36,37]. The immunohistochemical data in this study provide direct cytological evidence for their divergent roles. In the gills of the control group, the protein location of EcCAc was confined to the apical membrane of pillar cells, whereas EcCAg was evenly distributed throughout the pillar cells, and EcNKA predominantly occupied septal cells. This distinct distribution implies a sophisticated division of labor within the gill lamella. Septal cells, rich in NKA, likely serve as the primary sites for active Na^+^ pumping, while pillar cells, housing both CA isoforms, are strategically positioned to modulate local pH and supply H^+^ or HCO_3_^−^ for apical and basolateral ion exchangers [38,39]. Upon low-salinity stress, the protein location of EcCAc underwent a notable shift from the apical to the basolateral membrane of pillar cells, accompanied by an increase in fluorescence intensity. In hyper-regulating crustaceans exposed to dilute media, basolateral HCO_3_^−^ supply is essential for Na^+^/HCO_3_^−^ cotransporters (NBCs) and for maintaining intracellular pH during enhanced transcellular Na^+^ uptake [40,41]. The observed basolateral translocation of EcCAc likely represents a rapid adaptive response to augment basolateral proton-equivalent flux. In contrast, EcCAg concentrated at the apical membrane under both stress conditions, consistent with its proposed role in supplying H^+^ and HCO_3_^−^ for apical ion exchangers, such as Na^+^/H^+^ and Cl^−^/HCO_3_^−^ antiporters, to sustain transepithelial ion flux [3,42]. This functional segregation, with CAc mediating basolateral pH regulation and CAg mediating apical ion exchange, parallels the differential localization of CA isoforms in vertebrate renal tubules and fish gills, suggesting a conserved evolutionary strategy for epithelial ion transport [43,44].

The presence of two functionally distinct CA isoforms in the gills of *E. carinicauda* is consistent with findings in other decapods, including *Callinectes sapidus* and *Litopenaeus vannamei* [14,36]. However, our data add a temporal dimension by showing that these isoforms are not merely redundant duplicates but are deployed at different developmental stages. The early location of EcCAg in the epidermis and intestine of nauplii and zoea, preceding gill-specific expression of both CAs, suggests that EcCAg may represent a more ancestral or general-purpose isoform used by immature epithelia, whereas EcCAc becomes increasingly important as the specialized gill epithelium matures. This ontogenetic switch may mirror the evolutionary transition from integumentary to branchial respiration and ion regulation that occurred during the radiation of decapod crustaceans [39]. Moreover, the specific restriction of EcNKA to septal cells in the gill, rather than pillar cells, distinguishes *E. carinicauda* from some other penaeid shrimps where NKA is found in pillar cells. This cell-type specificity may reflect a narrower ion-transport pathway in *E. carinicauda*, where septal cells are primarily responsible for active ion transport and pillar cells serve to regulate pH homeostasis. The functional consequences of this anatomical arrangement warrant further investigation using high-resolution imaging and electro-physiological approaches.

### 4.3. Transcriptional and Post-Translational Control Under Salinity and Alkalinity Stress

*EcNKA*, *EcCAc* and *EcCAg* are pivotal genes involved in ion transport and acid–base regulation, playing critical roles in the adaptive responses of *E. carinicauda* to salinity and alkalinity stress. In the present study, however, it should be explicitly acknowledged that the HA treatment inevitably introduced a concomitant pH increase (from 8.0 to ~8.5) due to NaHCO_3_ addition, rendering alkalinity and pH non-separable confounders in this treatment. With this caveat in mind and given that the alkalinity elevation (to 12 mmol/L) was substantially more drastic than the mild pH shift, we primarily attribute the observed responses to alkalinity stress while recognizing the potential minor synergistic contribution of elevated pH. Below, the specific transcriptional and protein-level responses of these three genes under low-salinity and high-alkalinity stress are detailed. A noteworthy aspect of this study is the discordance between mRNA expression and protein-level responses. The expression of *EcNKA* showed no significant difference compared with the control group under low-salinity stress, while it was significantly downregulated under high-alkalinity stress. At the protein level, the mean fluorescence intensity of EcNKA decreased under high-alkalinity stress, whereas no significant change was observed under low-salinity stress. This pattern is largely consistent with its transcriptional level. This concordance suggests that, for EcNKA, changes in transcript abundance are directly reflected at the protein level, indicating that transcriptional regulation serves as the primary determinant of its functional capacity under these stress conditions. The decrease in EcNKA protein under high alkalinity is particularly striking, as it contrasts with the transcriptional upregulation reported in previous studies on *E. carinicauda* [22,23]. This suggests that high carbonate alkalinity may exert direct inhibitory effects on NKA protein stability or promote its degradation, possibly through interference with the enzyme’s conformational state or via increased oxidative stress. Such post-translational downregulation may represent an energetic trade-off: under alkaline stress, the shrimp may prioritize acid–base balance rather than active ion uptake, conserving ATP for pH regulation via CA-mediated pathways.

In addition, under both low-salinity and high-alkalinity stress, *EcCAc* transcripts were upregulated, while *EcCAg* transcripts were significantly downregulated. At the protein level, EcCAc underwent membrane translocation with increased fluorescence intensity under low-salinity and high-alkalinity stress, and EcCAg showed apical concentration with decreased fluorescence intensity and area under low-salinity stress. These discrepancies in transcriptional levels and protein levels of EcCAc and EcCAg indicate that transcriptional changes do not simply translate to corresponding protein deployment, and that post-translational mechanisms, such as phosphorylation, palmitoylation, or interactions with scaffolding proteins, play pivotal roles in modulating ion transport capacity [25,45,46].

## 5. Conclusions

In summary, the osmoregulatory apparatus of *E. carinicauda* is progressively established from the zoea stages, with the gill epithelium becoming the predominant site for EcNKA, EcCAc and EcCAg expression at the postlarval stage. Within the gills, EcNKA localizes to septal cells, whereas EcCAc and EcCAg localize to pillar cells with distinct membrane distributions, revealing cell-type-specific functional segregation. Low-salinity and high-alkalinity stresses induce both transcriptional changes and subcellular redistributions of these proteins, and the discordance between mRNA levels and protein localization of EcCAc and EcCAg underscores the importance of post-translational trafficking in modulating ion-transport capacity. Together, these findings reveal a cytological link between the developmental maturation of osmoregulation and saline–alkaline adaptation in *E. carinicauda*.

## Data Availability

Data are contained within the article and Supplementary Materials.

## Supplementary Materials

The following supporting information can be downloaded at https://www.mdpi.com/article/10.3390/biology15171554/s1: Table S1: Primers used in this study; Table S2: ELISA results of pre-immune serum and purified antibody of EcCAc; Table S3: ELISA results of pre-immune serum and purified antibody of EcCAg. Figure S1: SDS-PAGE (A) and Western blot analysis (B) of EcCAc protein; Figure S2: SDS-PAGE (A) and Western blot analysis (B) of EcCAg protein; Figure S3: Specificity detection of anti-NKA (A), anti-EcCAc (B) and anti-EcCAg (C) polyclonal antibodies in the gills of *E. carinicauda*.

## Abbreviations

The following abbreviations are used in this manuscript:

CA	carbonic anhydrase
CAc	cytoplasmic carbonic anhydrase
CAg	glycosylphosphatidylinositol-linked carbonic anhydrase
IHC	immunohistochemistry
IS	immunostaining
LS	low-salinity
HA	high-alkalinity
N	nauplius
NKA	Na^+^/K^+^-ATPase
PBS	phosphate-buffered saline
PFA	paraformaldehyde
PL5	postlarva at day 5
PZ	pre-zoea
qRT-PCR	quantitative real-time PCR
ZI-ZV	zoea I to zoea V
