# Supplementary material for: Ontogeny and Stress-Induced Plasticity of Osmoregulatory Machinery in Exopalaemon carinicauda: Distinct Spatiotemporal Localization of Na+/K+-ATPase and Carbonic Anhydrase Isoforms
Source: Biology (Basel). 2026 Sep 5;15(17):1554. doi: 10.3390/biology15171554 (PMC13565046; doi:10.3390/biology15171554)
Supplement: Supplementary file 1 [file biology-15-01554-s001.zip › biology-4508058-supplementary.pdf]

Table S1. Primers used in this study

Purpose	Forward primer (5′→3′)	Reverse primer (5′→3′)
qPCR for *EcNKA*	TCTTCCTCATTGCCTTCATCCTTG	ACATACAGTGACAGTAGCCAACAG
qPCR for *EcCAc*	GAAGCTGAACCGTCTCCAAG	GGACCTCCAGTTAGGCATGA
qPCR for *EcCAg*	AACGAAGTCGTCACCTGGAC	GTGGACGGAAGTTGTTGACC
qPCR for 18s rRNA	TATACGCTAGTGGAGCTGGAA	GGGGAGGTAGTGACGAAAAAT
Prokaryotic expression cloning for *EcCAc*	CCGGAATTCATGGTTTGGGGATACGCC	ATAAGAATGCGGCCGCTTATTCTTCTTCTTGTGAG
Prokaryotic expression cloning for *EcCAg*	CCGGAATTCCCGGCTTGGACTTACACTGGAC	ATAAGAATGCGGCCGCCATGTTCATCATGAGGACGGCC

Note: The sequences underlined indicate the 5′ protective bases and restriction endonuclease recognition sequences.

Table S2. ELISA results of pre-immune serum and purified antibody of EcCAc

**Concentration (ng/ml)**	**NC**	**1,000.00**	**500.00**	**250.00**	**125.00**	**62.50**	**31.25**	**15.62**	**7.81**	**3.90**	**1.95**	**Blank**	**/**
**Dilution**	1:1,000	1:1,000	1:2,000	1:4,000	1:8,000	1:16,000	1:32,000	1:64,000	1:128,000	1:256,000	1:512,000	Blank	Titer
**Antibody**	0.091	2.579	2.546	2.590	2.532	2.416	2.322	2.164	1.785	1.350	0.909	0.051	>1:512,000

Table S3. ELISA results of pre-immune serum and purified antibody of EcCAg

**Concentration (ng/ml)**	**NC**	**1,000.00**	**500.00**	**250.00**	**125.00**	**62.50**	**31.25**	**15.62**	**7.81**	**3.90**	**1.95**	**Blank**	**/**
**Dilution**	1:1,000	1:1,000	1:2,000	1:4,000	1:8,000	1:16,000	1:32,000	1:64,000	1:128,000	1:256,000	1:512,000	Blank	Titer
**Antibody**	0.132	2.542	2.420	2.529	2.395	2.298	2.148	1.940	1.710	1.328	0.991	0.143	>1:512,000

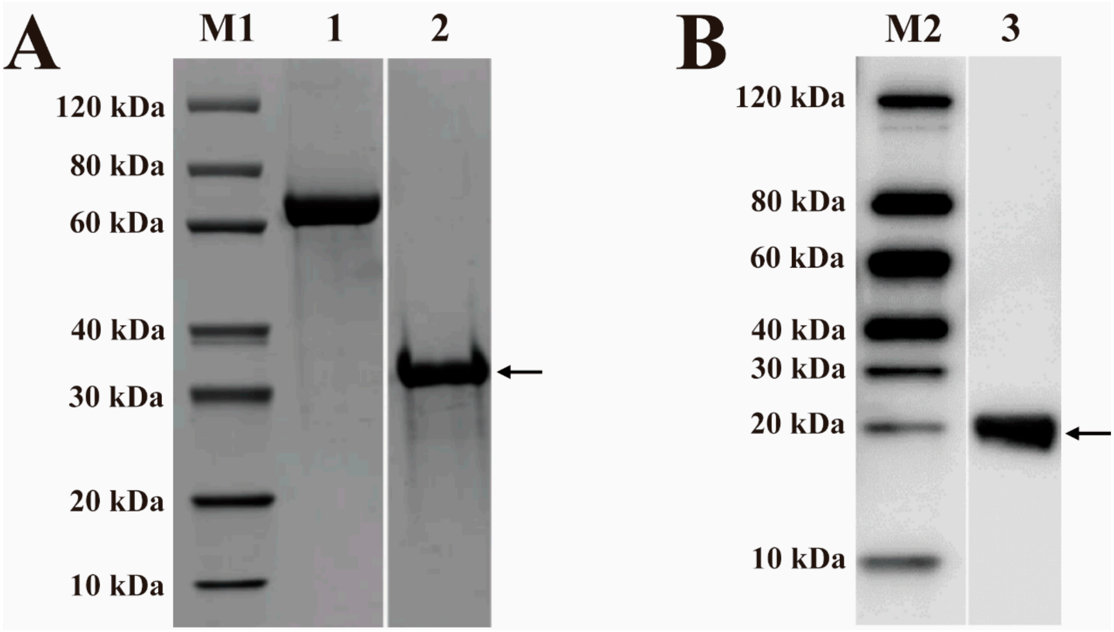

Figure S1. SDS-PAGE (A) and Western blot analysis (B) of EcCAc protein. Lane M1 and M2, Protein Marker. Lane 1, BSA (5.00 μg); Lane 2, EcCAc (5.00 μg); Lane 3, EcCAc protein.

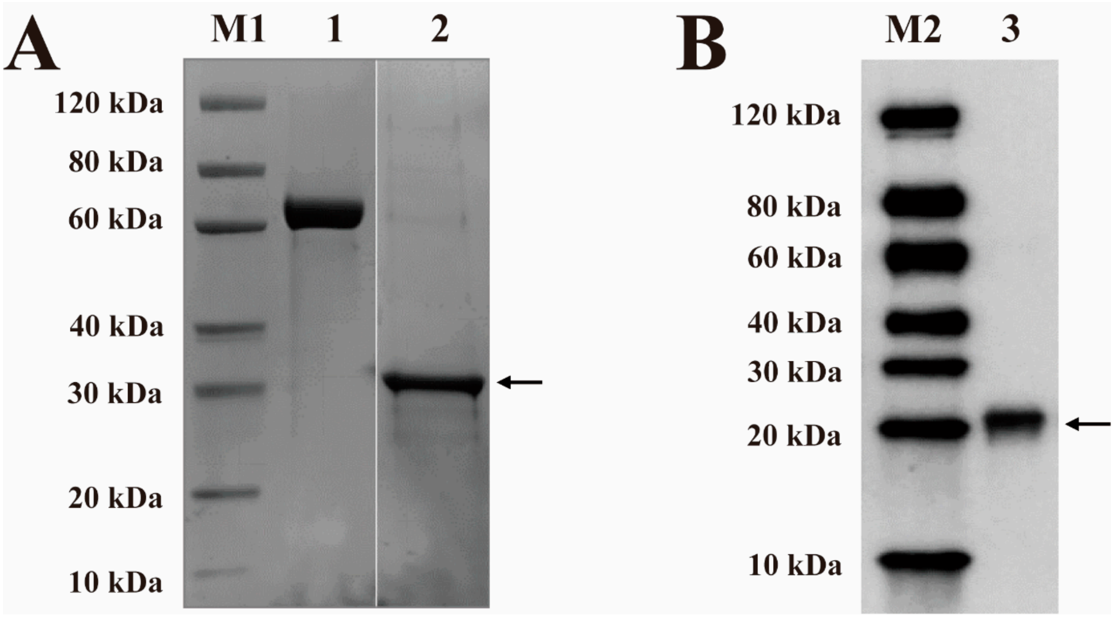

Figure S2. SDS-PAGE (A) and Western blot analysis (B) of EcCAg protein. Lane M1 and M2, Protein Marker; Lane 1, BSA (5.00 μg); Lane 2, EcCAg (5.00 μg); Lane 3, EcCAg protein.

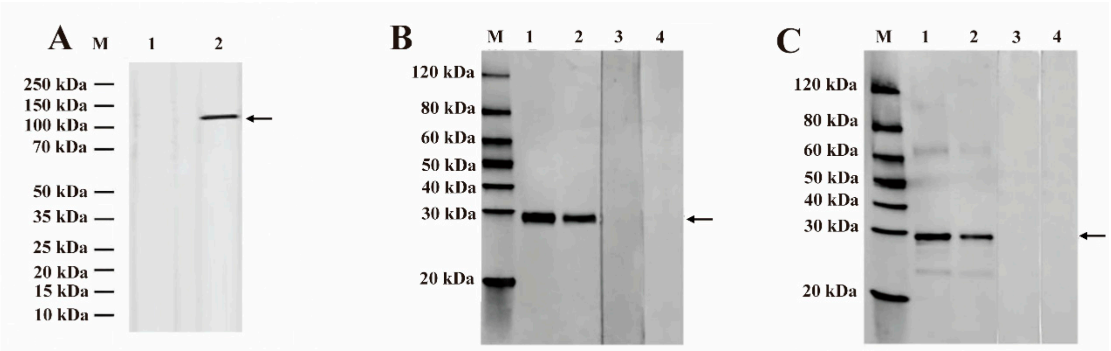

Figure S3. Specificity detection of anti-NKA (**A**), anti-EcCAc (**B**) and anti-EcCAg (**C**) polyclonal antibody in the gills of *E. carinicauda*. (A) Lane 1, pre-serum of anti-NKA; Lane 2, 100 ng anti-NKA. (B) Lane 1, 100 ng purified antibody of EcCAc; Lane 2, 50 ng purified antibody of EcCAc; Lane 3, pre-serum of EcCAc; Lane 4, blank. (C) Lane 1, 100 ng purified antibody of EcCAg; Lane 2, 50 ng purified antibody of EcCAg; Lane 3, pre-serum of EcCAg; Lane 4, blank. M, Protein Marker. The arrow points to the position of the target protein band.
